# The Effects of circ_000558/miR-1225-5p/ARL4C on Regulating the Proliferation of Renal Cell Carcinoma Cells

**DOI:** 10.1155/2023/1303748

**Published:** 2023-02-02

**Authors:** Ling Xia, Minghuan Ge, Guang Shan, Huijun Qian, Yue Xia

**Affiliations:** ^1^Department of Radiation and Medical Oncology, Zhongnan Hospital of Wuhan University, Wuhan 430071, Hubei, China; ^2^Department of Urology, Renmin Hospital of Wuhan University, Wuhan 430060, Hubei, China

## Abstract

Renal cell carcinoma (RCC) is one of the top ten tumors over the world. RCC is not sensitive to radiotherapy and chemotherapy. Therefore, it is necessary to find new targets for the treatment. CircRNAs are a special type of noncoding RNAs, which play important roles in many types of cancer. In this study, we found circ_000558 was upregulated in RCC cells, and it elevated the proliferation ability of RCC cells. The relationship between miR-1225-5p and circ_000558 or ARL4C was predicted via circBank and circular RNA interactome and confirmed by dual-luciferase reporter assay. Then, the effects of circ_000558/miR-1225-5p/ARL4C on RCC cell proliferation and apoptosis were assessed by CCK-8 assay. The results revealed that the knockdown of ARL4C significantly reduced RCC cell proliferation and overexpression of circ_000558 could significantly induce RCC cell proliferation after miR-1225-5p treatment further promoted the inhibitory ability of ARL4C knockdown. Overall, our study suggested that circ_000558/miR-1225-5p/ARL4C network was related to the RCC cell proliferation. This finding could provide new targets for the treatment and prognosis of RCC.

## 1. Introduction

Renal cell carcinoma (RCC) is the most common malignant tumor of renal parenchyma, which is one of the top ten tumors in the world, accounting for 3.7% of all new cancer cases in recent years [1–3]. Despite the continuous improvement of diagnostic techniques, one-third of the patients still exert local progression or distant metastasis at the time of initial diagnosis, and about one-fourth of the patients with localized lesions or with feasible organ resection eventually developed metastatic disease [4–6]. The median time of recurrence after surgery was 1.9 years [7]. RCC is not sensitive to radiotherapy and chemotherapy, and the current treatment options include surgical resection, immunotherapy, targeted therapy, radiotherapy, and chemotherapy. Surgery is the main treatment strategy for RCC, especially the localized renal cell carcinoma [8, 9]. For patients with locally advanced and metastatic renal cell carcinoma [10], systematic treatment is required in addition to surgery to reduce the recurrence or prolong the survival of patients [11, 12]. Therefore, it is very necessary to explore the novel occurrence, development, and prognostic mechanism of RCC and to develop new targets for the treatment and prognosis assessment of RCC.

Noncoding RNAs are a group of RNAs with gene expression regulation functions and without gene coding functions [13]. Noncoding RNAs mainly contain long noncoding RNAs, such as circRNAs [14] and lncRNAs [15]. Short noncoding RNAs mainly refer to microRNAs (miRNAs) [16]. CircRNA is a special type of noncoding RNA, which has no free end and is difficult to be degraded by exonuclease. It has a covalently bonded 3′ and 5′ ends to form a covalently closed circular structure [17, 18]. Many studies have shown that circRNAs exist in large and stable quantities in eukaryotes, are involved in various cell biological activities and functions such as protein synthesis, gene expression, and post-transcriptional modification, and play a very important regulatory role [19–21]. Besides, many studies have reported that circRNAs play a crucial role in the pathogenesis of various tumors [22, 23].

MiRNAs are a group of short noncoding RNA [24]. CircRNAs are reported to exert their function via inhibiting the regulation role of miRNAs on their target genes. MiRNAs have been widely investigated in many tissues and diseases, such as bone marrow stem cells [25], neurons [26], intervertebral disc [14], and RCC [27]. In renal cancer, circ_001895 and circ_RNRIP1 can regulate cell proliferation and promote the occurrence of renal cancer [28, 29]. However, the mechanism of circRNA in regulating the development of RCC is still unclear. In this study, we focus on the mechanism of circ_000558 on the proliferation of RCC cells.

## 2. Materials and Methods

### 2.1. Cell Culture and Transfection

Human RCC cells (ACHN, 786-O, 769-P, and Caki-1) and human renal epithelial cells (HK2) were purchased from the ATCC (Manassas, VA, USA). Cells were cultured in Roswell Park Memorial Institute (RPMI) 1640 medium (Gibco, USA), supplemented with 10% fetal bovine serum (FBS) (Gibco, USA) and maintained at 37°C and 5% CO_2_ incubator (Thermo, USA).

The plasmid for circ_000558 overexpression, the small interference RNAs (siRNAs) for circ_000558 and ARL4C knockdown, the miR-1225-5p mimics, inhibitor, and negative control (NC) were synthesized by RiboBio company (Guangzhou, China). After seeded into plates, the cells were transfected by using Lipofectamine 2000 reagent (Life Technologies, Carlsbad, CA) according to the manufacturer's protocol [30].

### 2.2. Quantitative PCR (qPCR)

TRIzol reagent (Invitrogen, Life Technologies, Grand Island, USA) was used to extract total RNA of cells according to the previous study [31, 32]. After extraction, 5 *μ*g of total RNAs were incubated with 15-unit RNase R for 15 min at 37°C. circRNAs, mRNA, and miRNA expression was detected by SYBR™ Green PCR Master Mix (Bio-red, USA) on the ABI 7900HT (Applied Biosystems, Foster City, CA). We normalized and analyzed the relative expression data by using the equation 2^−ΔΔCt^ [33].

To identify the expression location of circ_000558, we isolated the nuclear and cytoplasmic fractions by NE-PER™ Nuclear and Cytoplasmic Extraction Reagents (Thermo, USA) following the manufacturer's instruction. The expression of circ_000558 in nuclear and cytoplasmic was measured and U6 snRNA and 18S rRNA were used as positive control for nuclear and cytoplasmic fractions, respectively.

### 2.3. Cell Viability

Cells were seeded at a density of 1500 cells/well in 96-well plates and transfected by oligo and plasmid. Then, we added 10 *μ*L of Cell Counting Kit‐8 (CCK‐8, Beyotime, Nantong, China) reagent into each well, and the cells were maintained at 37°C for 2 h. The absorbance (450 nm) was measured by an enzyme‐linked immunosorbent assay reader (Tecan, Männedorf, Switzerland) [34].

### 2.4. Apoptosis Assay

Flow cytometry was used to detect the cell apoptosis. The cells were stained by Annexin V-FITC Apoptosis Detection Kit (Beyotime, Hangzhou, China) according to the manufacturer's protocol. The samples were further measured by flow cytometer (FACScan, BD Biosciences, USA). The data were analyzed by Cell Quest software (BD Biosciences).

### 2.5. Dual-Luciferase Reporter Assay

The sequences of wild-type of circ_000558 and ARL4C 3′-UTR and mutation of circ_000558 and ARL4C 3′-UTR containing binding sites for miR-1225-5p were constructed. MiR-1225-5p mimics or miRNA NC with the plasmid were cotransfected into the cells. After transfection for 48 h, we detected the firefly and renilla luciferase activities using the Dual Glo Luciferase Assay System (Promega, WI, USA) according to the manufacturer's protocol [35].

### 2.6. Western Blot Assay

Total proteins were extracted using RIPA Lysis Buffer supplemented with PMSF (Beyotime, Hangzhou, China) as previously described [36, 37]. The concentration of total proteins was determined by a Bradford protein assay (Beyotime, Hangzhou, China). Equal protein was loaded and separated by SDS-PAGE and transferred onto a polyvinylidene difluoride (PVDF) membrane (Millipore, USA), and then it was blocked with 5% nonfat milk. The membrane was incubated with primary antibodies (ARL4C, Bcl-2, Bax, and cleaved Caspase-3) at 4°C overnight. After washing with PBS, the membrane was incubated with secondary antibodies at room temperature for 1 h. The band was exposed by BeyoECL Plus (Beyotime, Hangzhou, China) and analyzed by Quantity One software (Bio-Rad, San Diego, CA, USA).

### 2.7. Tumor Xenograft in Nude Mice

Male nude BALB/C mice (4 weeks old) weighed 16–20 g were purchased from the Laboratory Animal Centre, Wuhan University. ACHN cells (1 × 10^6^) were injected into the right axillary region of the nude mice. The mice were randomly divided into 3 groups: control, siRNA-circ_000558 (si-circ), and si-circ + miRNA-mimics. 10 *μ*g si-circ or miRNA-mimics were directly administered via intragastric injection into si-circ group or si-circ + miRNA-mimics group mice after 7, 9, 11, 14, and 16 days of tumor growth, respectively. PBS was used as a vehicle control. The tumor volume was measured every two days for tumor formation using calipers. After four weeks, the mice were sacrificed by exposure to carbon dioxide for 10 min after they were anaesthetized with isoflurane. Tumors were weighted and photographed. All animal experimental procedures were conducted in accordance with Chinese laws on experimental animals and approved by the Ethics Committee of Wuhan University.

### 2.8. Statistical Analysis

Graphpad prism 8.0 software (GraphPad Software Inc., La Jolla, CA) was used to analyze the data. All experiments were repeated three times. Data were showed as mean ± SD. Student's *t*-test was used for analyzing the differences between two groups. When *P* < 0.05, the data were considered as statistically significant.

## 3. Results

### 3.1. circ_000558 Was Highly Expressed in RCC Tissues and Cells

**Figure 1:**
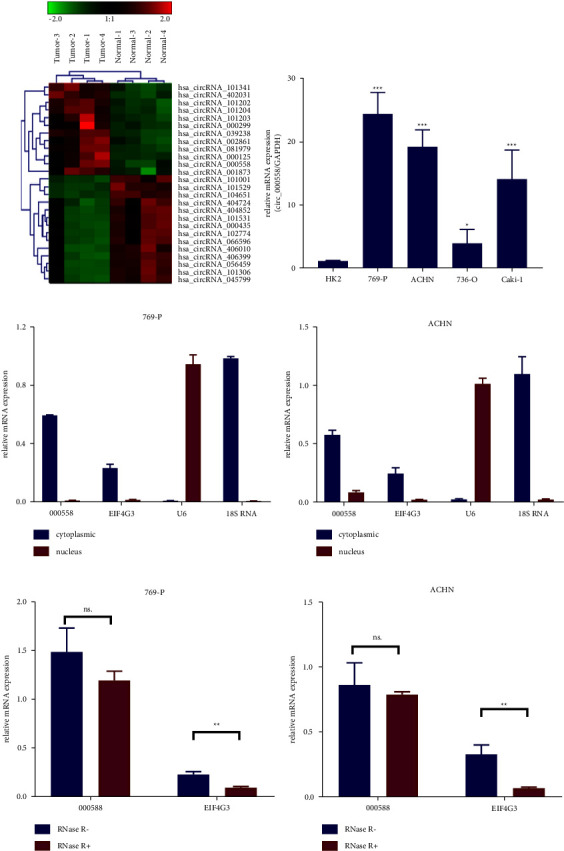
The expression of circ_000558 in RCC cells. (a) Hot map showed the differential expressed circRNAs between RCC tissues and matched nontumor tissues in GSE100186. (b) The expression of circ_000558 in RCC cells (ACHN, 786-O, 769-P, and Caki-1) and human renal epithelial cells (HK2) was tested by qPCR. ^*∗*^*p* < 0.05 and ^*∗∗∗*^*p* < 0.001 compared to HK2 cells. (c, d) The expression location of circ_000558 in 769-P (c) and ACHN cells (d) was tested by qPCR analysis. (e, f) The expression of circ_000558 in 769-P cells (e) and ACHN cells (f) after RNase R digested was tested by qPCR assay. ^*∗∗*^*p* < 0.01; ns, no significant difference.

We first analyzed the differential expression circRNAs in RCC tissues and matched nontumor tissues in GSE100186 [38]. We found that hsa_circ_000558 (circ_000558, also named as hsa_circEIF4G3_054) was significantly upregulated in RCC (Figure1(a)). To confirm the results of microarray, we measured the expression of circ_000558 in RCC cells (ACHN, 786-O, 769-P, and Caki-1) and human renal epithelial cells (HK2) by qPCR analysis (Figure 1(b)). Compared with HK2 cells, the expression of circ_000558 was much higher in RCC cells (ACHN, 786-O, 769-P, and Caki-1) (Figure 1(b)). Further studies showed that circ_000558 was mainly expressed in the cytoplasm (Figures 1(c) and 1(d)). To further confirm the existence of circ_000558, we preformed the RNase R resistant experiments (Figures 1(e) and 1(f)). The results showed that the circ_000558 was resistant to RNase R digestion (Figures 1(e) and 1(f)). The above results showed that circ_000558 was highly expressed in RCC tissues and cells.

### 3.2. Knockdown of circ_000558 Suppressed the Proliferation of RCC Cells

**Figure 2:**
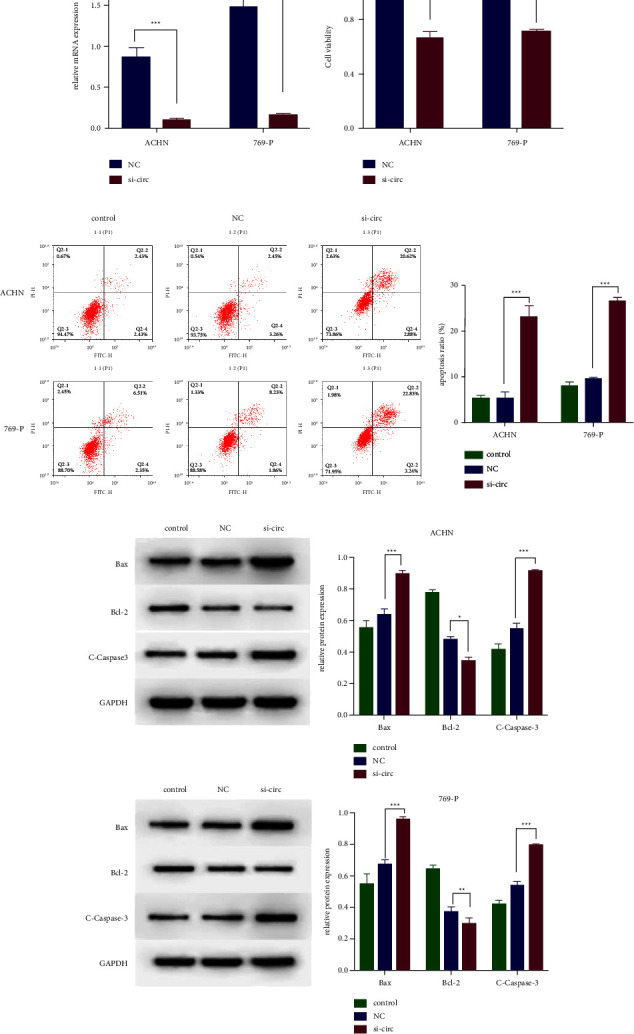
The effects of circ_000558 on cell proliferation and apoptosis in RCC cells. (a) The expression of circ_000558 in 769-P and ACHN cells after transfected with si-NC and si-circ_000558 (si-circ) was tested by qPCR analysis. (b) The inhibitor rate of cell proliferation in 769-P and ACHN cells after si-NC and si-circ transfection was measured using CCK-8 assay. (c) The cell apoptosis in 769-P and ACHN cells after si-NC and si-circ transfection was detected by flow cytometry. (d) The protein expression of apoptosis related protein (Bcl-2, Bax, and cleaved caspase-3) was analyzed by western blot assay (^*∗*^*p* < 0.05, ^*∗∗*^*p* < 0.01, and ^*∗∗∗*^*p* < 0.001).

We preformed the knockdown experiments by the specific siRNA targeting circ_000558 (si-circ) (Figure 2(a)). Then, CCK-8 assay was used to test the cell inhibition rate, and flow cytometry was applied to analyzed the cell apoptosis of RCC cells. CCK-8 assay showed that knockdown of circ_000558 significantly inhibited the cell proliferation of RCC cells (Figure 2(b)). The results of apoptosis assay indicated that knockdown of circ_000558 significantly increased the apoptosis rate of RCC cells (Figure 2(c)). Western blot assay showed that the expression of Bax and cleaved Caspase-3 was markedly upregulated, and the expression of Bcl-2 was downregulated by circ_000558 knockdown (Figure 2(d)). The above results suggested that knockdown of circ_000558 suppressed the proliferation of RCC cells.

### 3.3. circ_000558 Regulated RCC Cell Proliferation by Targeting miR-1225-5p

**Figure 3:**
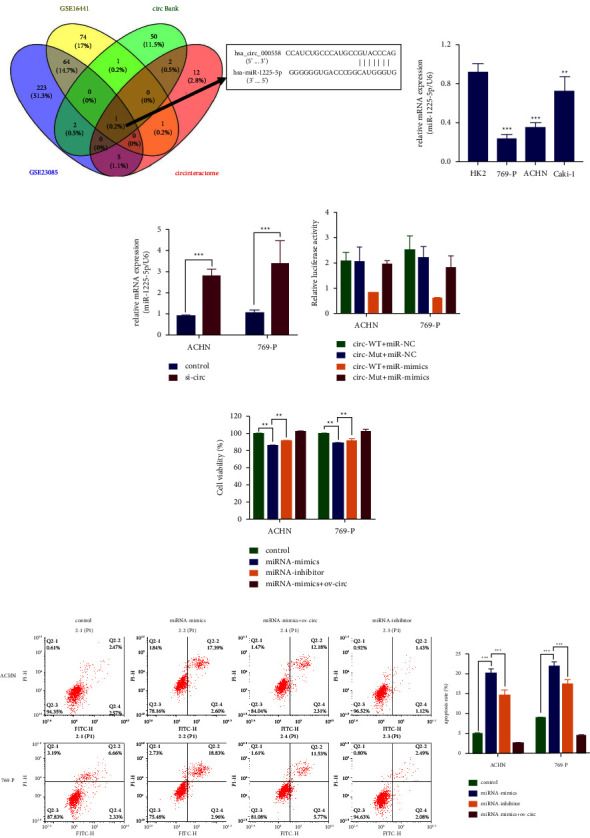
circ_000558 regulated RCC cell proliferation by inhibiting miR-1225-5p. (a) Venn diagram showed the overlapped potential target miRNAs in GSE23085, GSE16441, circBank, and circular RNA interactome. The binding site between miR-1225-5p and circ_000558 was shown. (b) The expression of miR-1225-5p in renal carcinoma cells (ACHN, 769-P, and Caki-1) and human renal epithelial cells (HK2) was tested by qPCR analysis. (c) The expression of miR-1225-5p in 769-P and ACHN cells after control and circ_000558 overexpression plasmid transfection was measured by qPCR. (d) The relative luciferase intensity in 769-P and ACHN cells after wild-type of circ_000558 (circ-WT) or mutation type of circ_000558 (circ-Mut) and miR-1225-5p mimics (miR-mimics) cotransfect was tested by dual-luciferase reporter assay. (e) The cell viability of 769-P and ACHN cells after miR-mimics, miR-inhibitor, or miR-mimics + ov-circ transfection was tested by CCK-8 assay. (f) The cell apoptosis of 769-P and ACHN cells after miR-mimics, miR-inhibitor, or miR-mimics + ov-circ transfection was tested by flow cytometry. (^*∗∗*^*p* < 0.01; ^*∗∗∗*^*p* < 0.001).

In this study, we aimed to investigate the underlying mechanism of circ_000558 in RCC. To find the potential target of circ_000558, we analyzed the downregulated miRNAs in RCC tissues in the public Gene Expression Omnibus (GEO) dataset GSE23085 [39] and GSE16441 [40], which is available from the website (https://www.ncbi.nlm.nih.gov/geo), and analyzed the predicted target miRNAs of circ_000558 by circBank and circular RNA interactome. Venn diagram showed that miR-1225-5p overlapped among the four databases (Figure 3(a)). qPCR results showed that the expression level of miR-1225-5p in RCC cells was lower than that in HK2 cells (Figure 3(b)), and it was increased after circ_000558 knockdown by qPCR (Figure 3(c)). Then, we further confirmed the relationship between circ_000558 and miR-1225-5p by dual-luciferase reporter assay. The results showed that the relative luciferase intensity was significantly reduced after transfected with wild-type of circ_000558 and miR-1225-5p mimics, and there was no significant alteration after mutation type of circ_000558 and miR-1225-5p mimics transfection (Figure 3(d)). Furthermore, we found that miR-1225-5p mimics could suppress the cell proliferation of RCC cells and miR-1225-5p inhibitor could significantly promote the cell proliferation (Figure 3(e)). Overexpression of circ_000558 could reduce the inhibitory effects of miR-1225-5p mimics on the cell proliferation of RCC cells (Figure 3(e)). Flow cytometry results showed that overexpression of circ_000558 could recover the apoptosis rate induced by miR-1225-5p mimics (Figure 3(f)). The results indicated that circ_000558 could regulate RCC cell proliferation by inhibiting miR-1225-5p.

### 3.4. The Effects of circ_000558/miR-1225-5p on *In Vivo* Growth of RCC Cells

**Figure 4:**
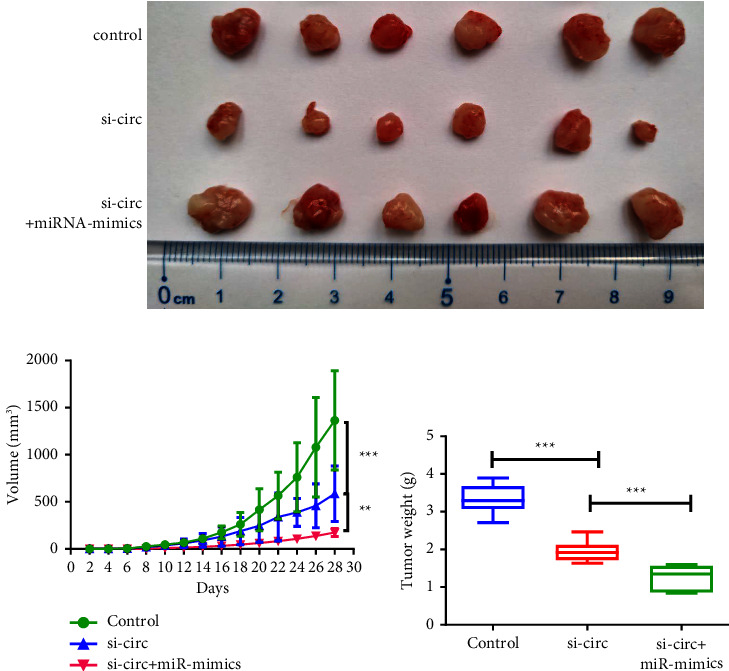
The effect of circ_000558/miR-1225-5p on RCC tumor growth *in vivo*. (a) The tumor tissues of control, si-circ, and si-circ + miR-mimics groups. (b) Tumor volume was measured every two days (*n* = 6). (c) Tumor weight was measured (^*∗∗*^*p* < 0.01, ^*∗∗∗*^*p* < 0.001).

We further evaluated the effect of circ_000558/miR-1225-5p on *in vivo* tumor growth in mice (Figure 4(a)). Knockdown of circ_000558 significantly inhibited tumor growth, and overexpression of miR-1225-5p significantly enhanced the inhibitory effects of circ_000558 knockdown on tumor growth (Figures 4(b) and 4(c)). The results indicated that circ_000558 regulated the tumor growth by targeting miR-1225-5p.

### 3.5. ARL4C Was a Potential Target of miR-1225-5p

**Figure 5:**
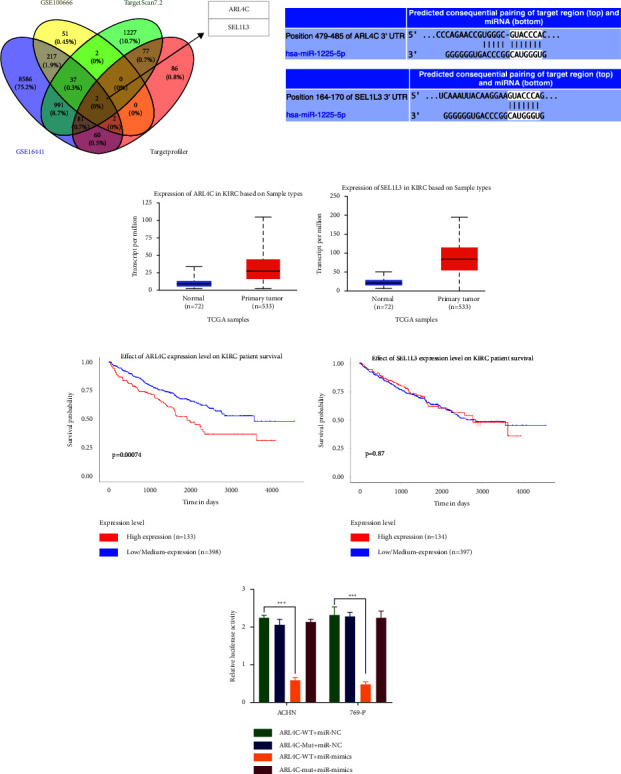
ARL4C was a potential target gene of miR-1225-5p. (a) Venn diagram showing the overlapped potential target genes in TargetScan7.2, Targetprofiler, GSE16441, and GSE100666. The binding site between miR-1225-5p, ARL4C, and SEL1L3. (b, c) The expression profile of ARL4C (b) and SEL1L3 (c) in RCC samples by TCGA analysis. Blue, normal samples; red, tumor samples. (d, e) Effect of ARL4C (d) and SEL1L3 (e) expression levels on renal carcinoma patients' survival; red, higher expression; blue, lower expression. (f) The relative luciferase intensity in 769-P and ACHN cells after wild-type of ARL4C (ARL4C-WT) or mutation type of ARL4C (ARL4C-Mut) and miR-1225-5p mimics (miR-mimics) cotransfect was tested by dual-luciferase reporter assay (^*∗∗∗*^*p* < 0.001).

To further investigate the function of miR-1225-5p, the potential target genes of miR-1225-5p were predicted by using TargetScan7.2 and Targetprofiler, and the upregulated genes in RCC tissues were analyzed in GSE16441 [41] and GSE100666. The results showed that two potential target genes (ARL4C and SEL1L3) were overlapped among the four databases (Figure 5(a)). Then, we analyzed the expression of ARL4C and SEL1L3 by TCGA analysis in UALCAN. The results showed that the expression of ARL4C and SEL1L3 was upregulated in RCC tissues (Figures 5(b) and 5(c)). ARL4C was significantly associated with overall survival (*p* = 0.00074), while SEL1L3 was not significantly associated with overall survival (*p* = 0.87) (Figures 5(d) and 5(e)). Thus, we chose ARL4C for further analysis. We confirmed the relationship between ARL4C and miR-1225-5p by using dual-luciferase reporter assay. The results showed that the luciferase intensity significantly reduced after wild-type of ARL4C 3′-UTR and miR-1225-5p mimics cotransfection, and there was no significant change after mutation type of ARL4C 3′-UTR and miR-1225-5p mimics (Figure 5(f)). These results revealed that ARL4C was a potential target of miR-1225-5p in RCC cells.

### 3.6. circ_000558/miR-1225-5p/ARL4C Regulated RCC Cell Proliferation

**Figure 6:**
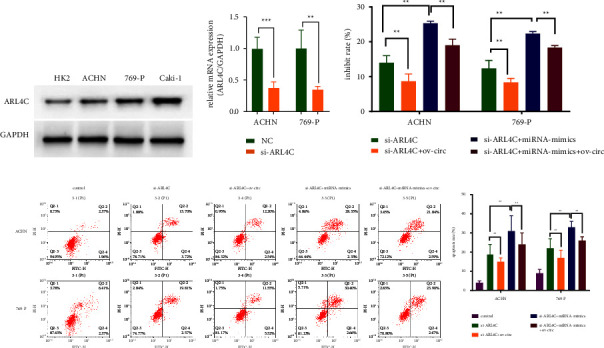
circ_000558/miR-1225-5p/ARL4C regulated RCC cell proliferation. (a) The protein expression of ARL4C in RCC cells (ACHN, 769-P, and Caki-1) and human renal epithelial cells (HK2) was tested by qPCR. (b) qPCR analysis was applied to assess the expression of ARL4C. (c) Cell inhibitor ratio of 769-P and ACHN cells after si-ARL4C, si-ARL4C + ov-circ, si-ARL4C + miR-mimics, or si-ARL4C + miR-mimics + ov-circ transfection was tested by CCK-8 assay. (d) The cell apoptosis of 769-P and ACHN cells after si-ARL4C, si-ARL4C + ov-circ, si-ARL4C + miR-mimics, or si-ARL4C + miR-mimics + ov-circ transfection was tested by flow cytometry. (^*∗∗*^*p* < 0.01 and ^*∗∗∗*^*p* < 0.001).

Then, we further investigated the roles of circ_000558/miR-1225-5p/ARL4C in cell proliferation of RCC cells. First, western blot assay showed that ARL4C was highly expressed in RCC cells (Figure 6(a)). Next, we found that the expression of ARL4C was significantly decreased after the cells transfected with si-ARL4C in RCC cells (Figure 6(b)). Furthermore, we found that knockdown of ARL4C significantly reduced cell proliferation and overexpression of circ_000558 could significantly reverse the reduced cell proliferation after ARL4C knockdown, which was recovered by miR-1225-5p mimics (Figure 6(c)). Meanwhile, knockdown of ARL4C significantly increased the cell apoptosis and overexpression of circ_000558 could significantly decrease the increased cell apoptosis after ARL4C knockdown, which could be recovered by miR-1225-5p mimics (Figure 6(d)). The results showed that circ_000558 could regulate the cell proliferation of RCC cells via miR-1225-5p/ARL4C pathway.

## 4. Discussion

CircRNAs have been originally regarded as by-products of aberrant splicing [42, 43]. Recently, a growing number of studies have shown that circRNAs are usually differentially expressed in different cancers and exert important roles in many cellular activities and exhibit a strong relationship with the development of cancers [44, 45]. For example, increasing evidences have demonstrated that circRNAs have been demonstrated to play important roles in RCC development [46–48]. In spite of several recent studies that have suggested their circRNA-miRNA-mRNA regulatory network in RCC, the current knowledge of circRNA-associated ceRNA network in RCC is still inadequate, which needs to be further investigated. In our study, hsa_circ_000558 was significantly upregulated in RCC tissues and cells, and knockdown of circ_0000558 significantly suppressed the cell proliferation of RCC cells. The result suggested that circ_0000558 was related to the occurrence and development of RCC. However, the roles of circ_000558 were still unclear and need further investigation.

Multiple studies have confirmed that CircRNAs always act as miRNAs inhibitor by sponging miRNAs. Therefore, potential miRNAs of circ_000558 were predicted in our study. We found that circ_000558 could bind with miR-1225-5p and then inhibit the expression of miR-1225-5p. In our study, overexpression of circ_000558 could significantly reduce the suppressive effects of miR-1225-5p on the cell proliferation of RCC. Thus, miR-1225-5p might be a potential target of circ_000558 on the cell proliferation of RCC.

miRNAs always act as a gene suppressor by binding to the 3′-UTR of target mRNAs [49, 50]. Thus, we predicted the potential target genes of miR-1225-5p in RCC cells. We found that miR-1225-5p could bind to the 3′-URT of ARL4C, which was upregulated in RCC cells. ARL4C belongs to the ADP-ribosylation factor (ARF)-like 4 protein subfamily (ARL4), which is a small GTP-binding (G) protein [51]. Recent studies demonstrated that ARL4C promotes the progression of lung cancers, colorectal cancers [52], and gastric cancers [53]. In RCC, upregulated expression of ARL4C is associated with poor prognosis and high possibility of metastasis [54]. According to the TCGA analysis, ARL4C was highly expressed in RCC tissues and significantly related with overall survival. Knockdown of ARL4C could significantly reduce the RCC cell proliferation and induce the cell apoptosis, which was restored by circ_000558 overexpression. Taking all these data together, we successfully constructed a novel circRNA-miRNA-mRNA network in RCC, which provided new insight into the underlying molecular mechanism of RCC. Our study proposed circ_000558/miR-1225-5p/ARL4 network in RCC, which might be applied to investigate the underlying mechanism of circRNA-miRNA-mRNA network in the occurrence and development of other cancers. However, more efforts need to be taken to identify the functions of the established network in RCC by performing further experiments. Besides, the diagnostic and prognostic values of RNAs in the established networks should be assessed by using plenty of clinical data of patients in the future, which help to develop potential diagnostic and prognostic biomarkers for RCC. In conclusion, the abnormally expressed circ_000558 inhibited the expression of miR-1225-5p and then upregulated the expression of its target gene, ARL4C, to promote the proliferation of RCC cells. The mechanism of circ_000558/miR-1225-5p/ARL4C might be associated with the development and occurrence of RCC. However, this supposition needs more experiments to demonstrate.

## Data Availability

The data used to support the findings of this study are available from the corresponding author upon request.
